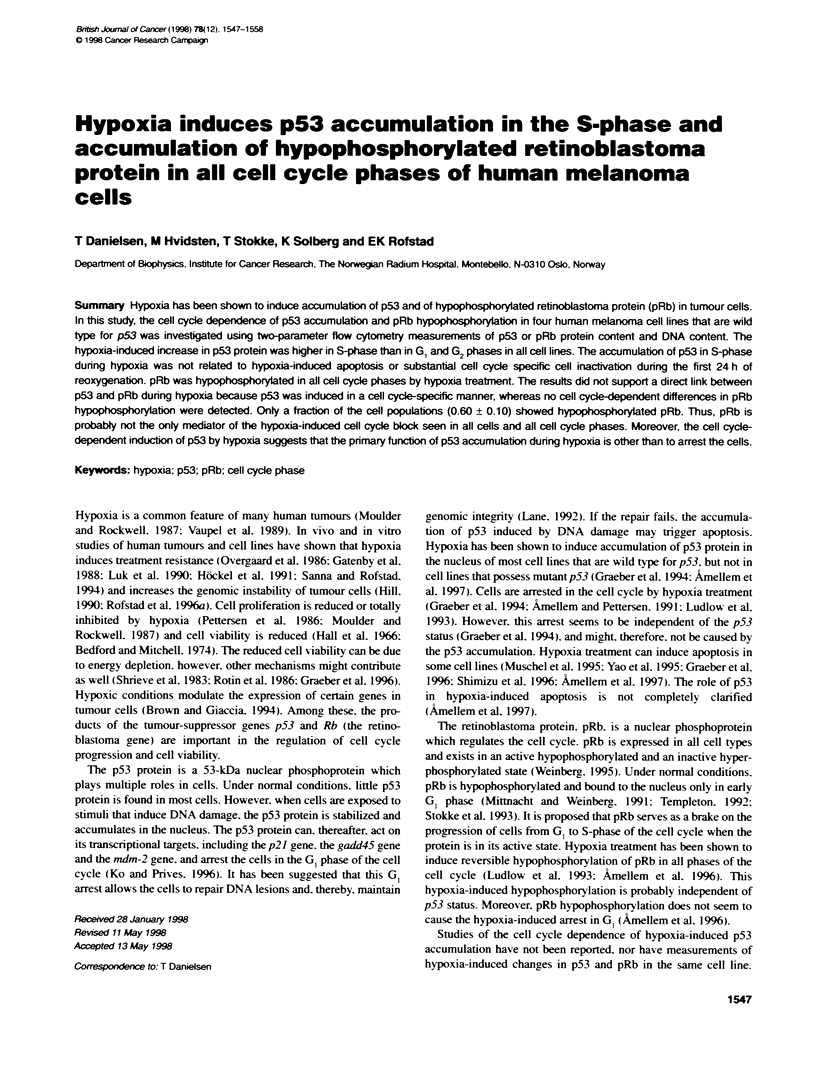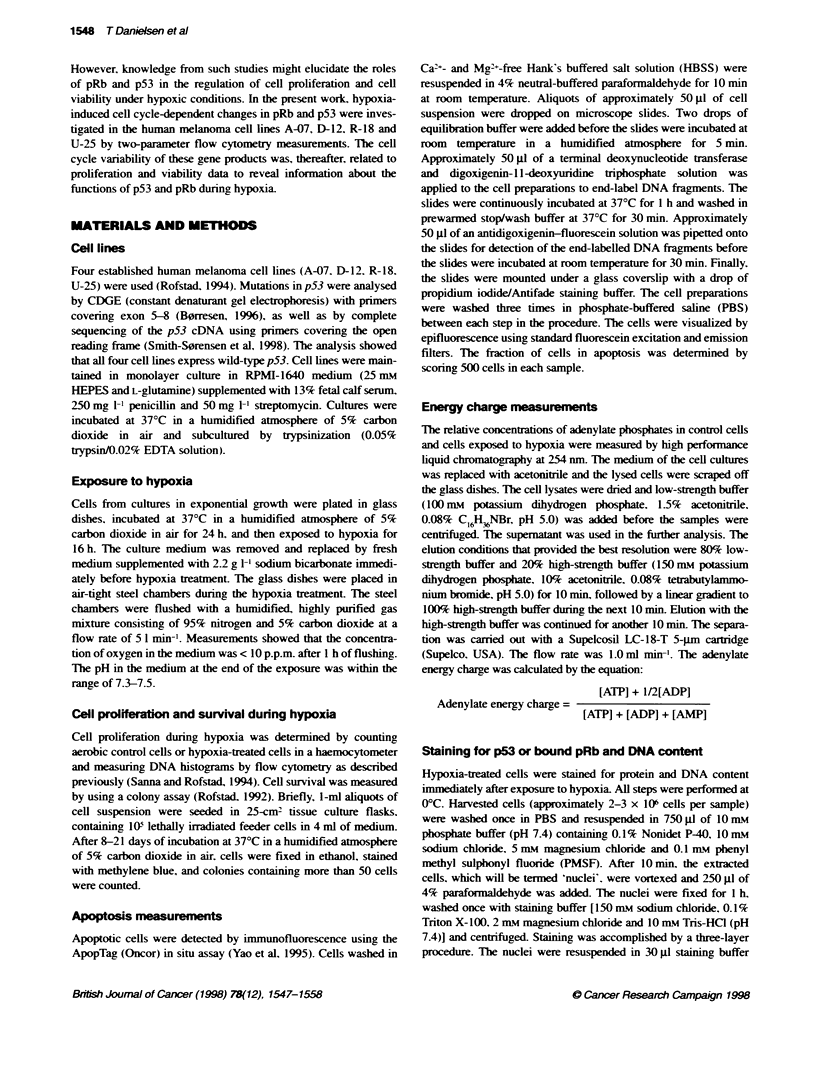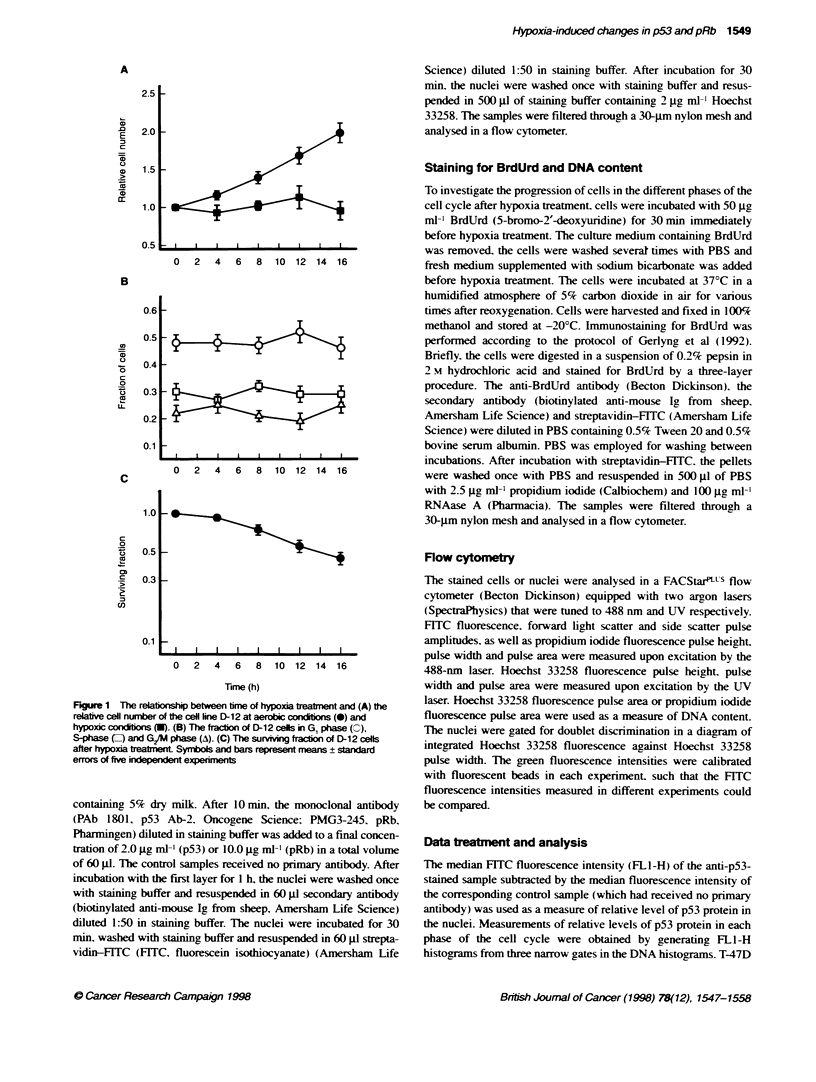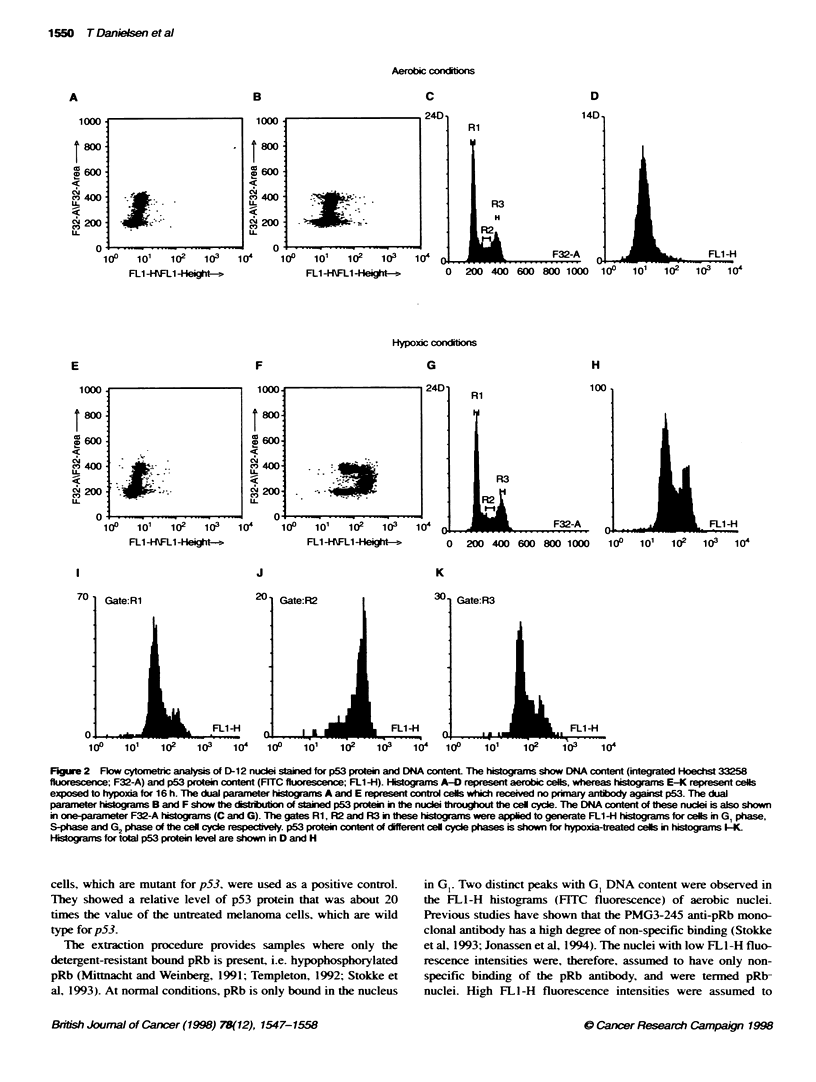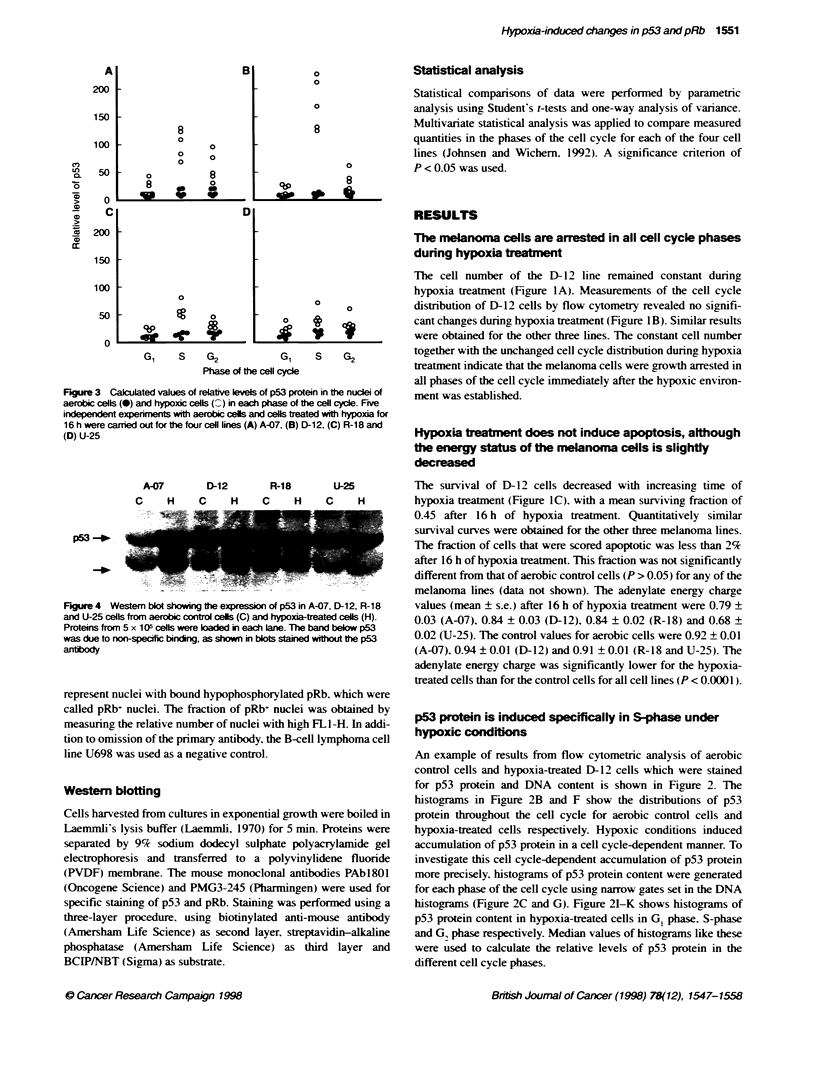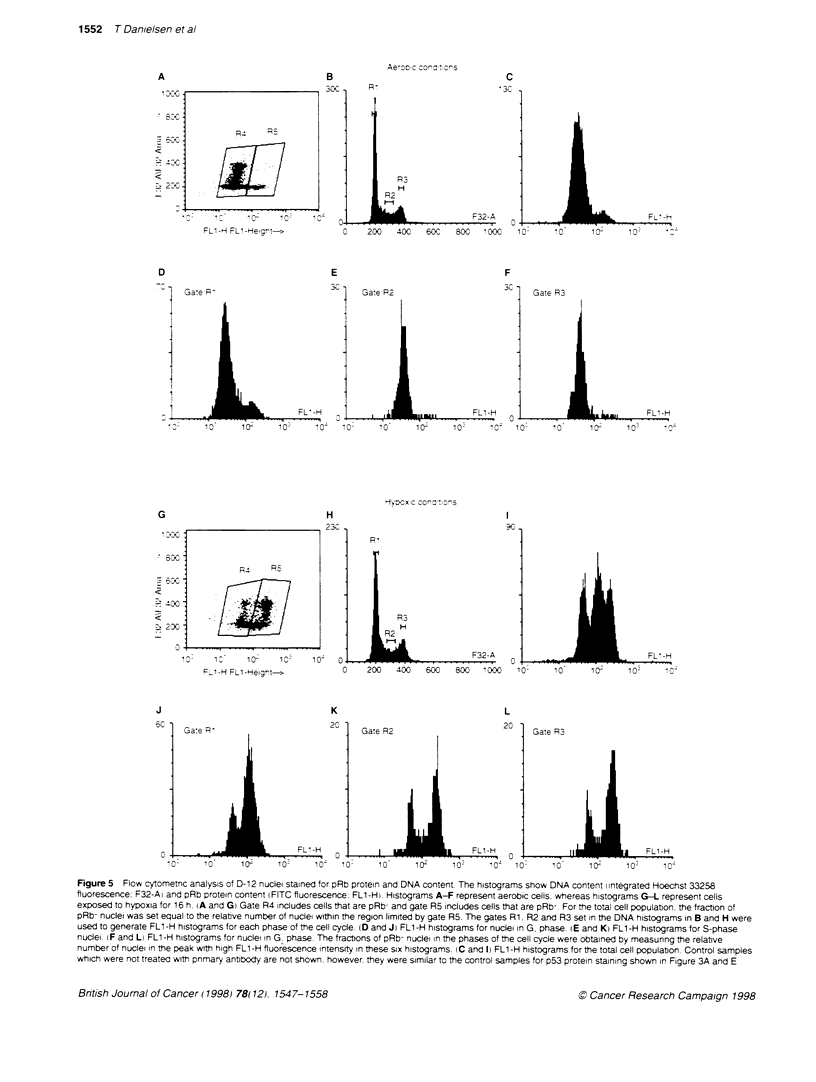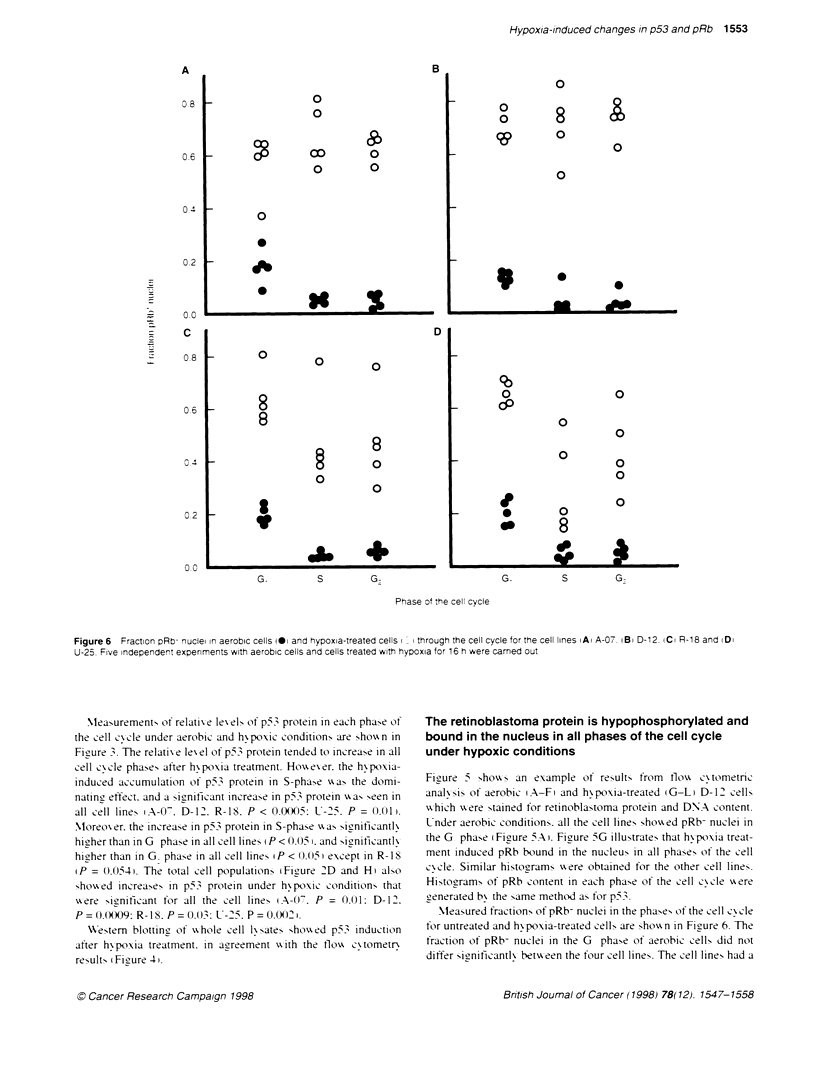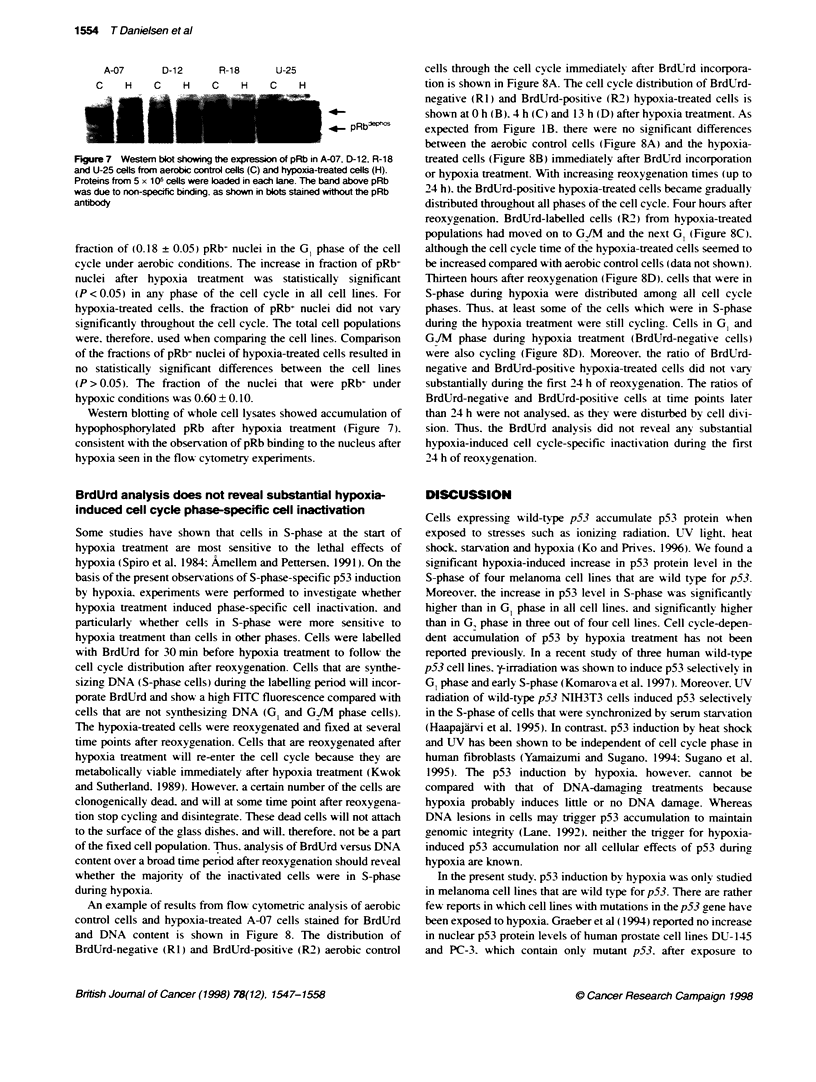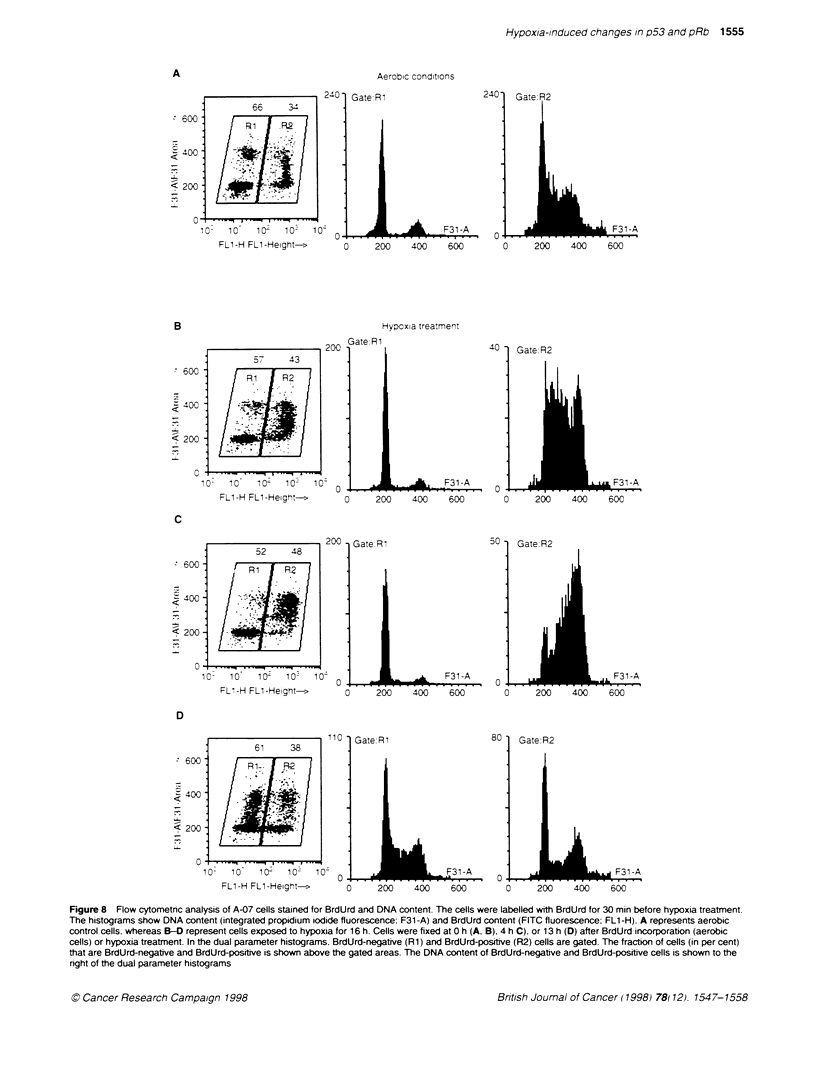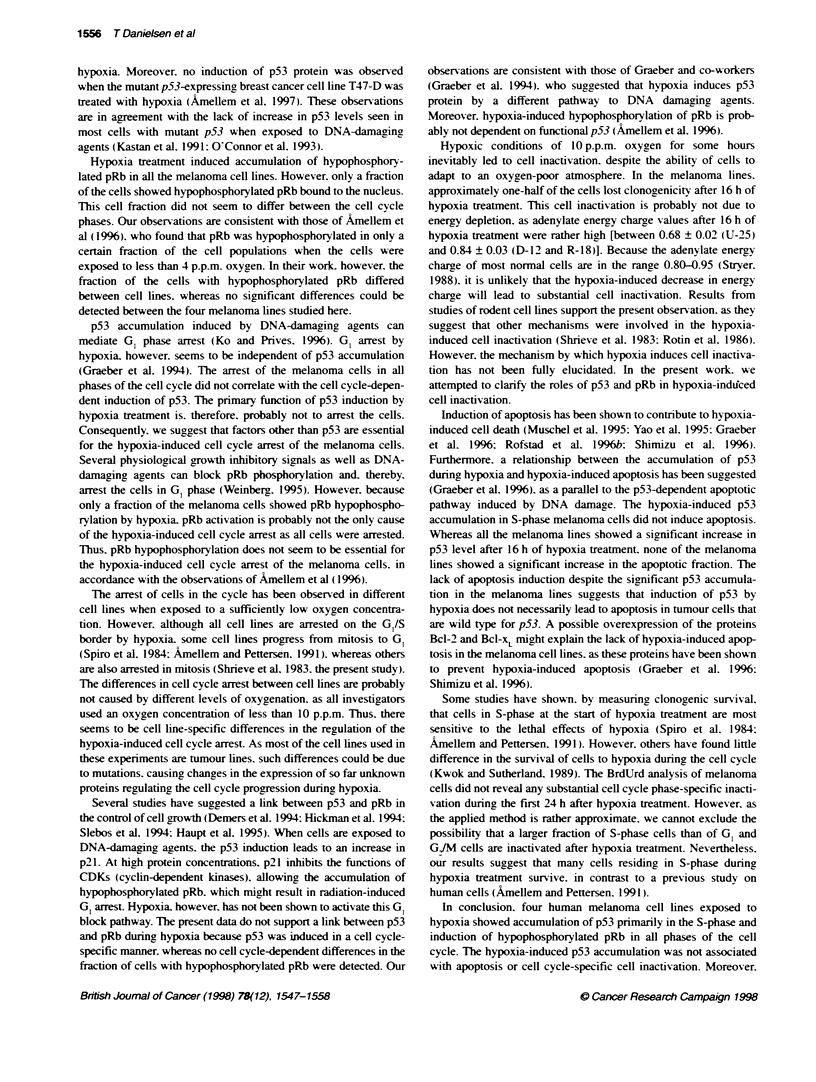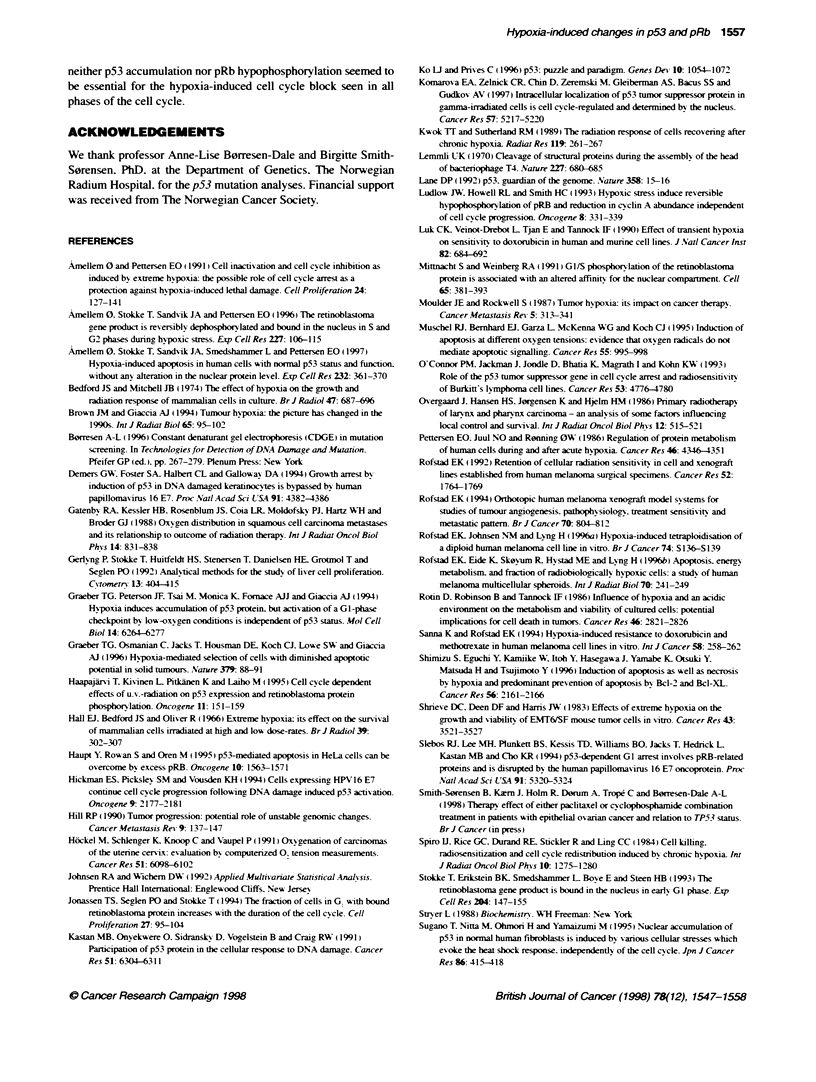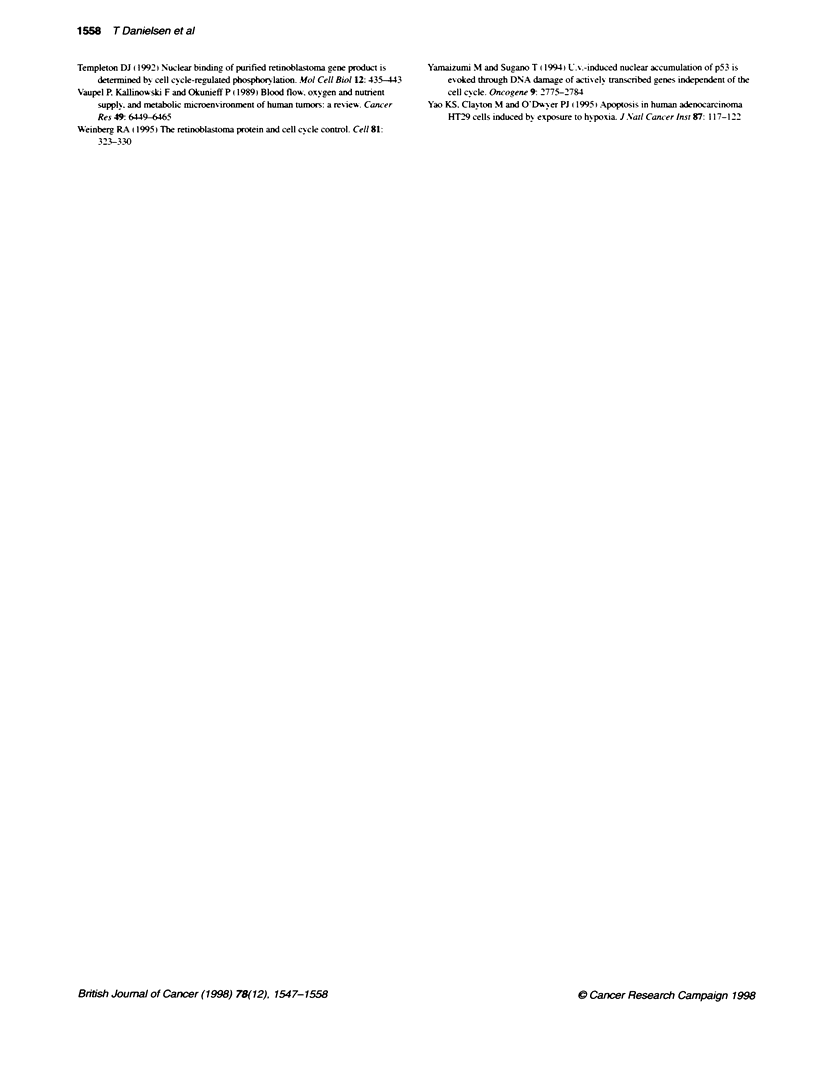# Hypoxia induces p53 accumulation in the S-phase and accumulation of hypophosphorylated retinoblastoma protein in all cell cycle phases of human melanoma cells.

**DOI:** 10.1038/bjc.1998.722

**Published:** 1998-12

**Authors:** T. Danielsen, M. Hvidsten, T. Stokke, K. Solberg, E. K. Rofstad

**Affiliations:** Department of Biophysics, Institute for Cancer Research, The Norwegian Radium Hospital, Montebello, Oslo, Norway.

## Abstract

**Images:**


British Journal of Cancer (1998) 78(12), 1547–1558
© 1998 Cancer Research Campaign

### Hypoxia induces p53 accumulation in the S-phase and accumulation of hypophosphorylated retinoblastoma protein in all cell cycle phases of human melanoma cells

**T Danielsen, M Hvidsten, T Stokke, K Solberg and EK Rofstad**

Department of Biophysics, Institute for Cancer Research, The Norwegian Radium Hospital, Montebello, N-0310 Oslo, Norway

**Summary** Hypoxia has been shown to induce accumulation of p53 and of hypophosphorylated retinoblastoma protein (pRb) in tumour cells. In this study, the cell cycle dependence of p53 accumulation and pRb hypophosphorylation in four human melanoma cell lines that are wild type for *p53* was investigated using two-parameter flow cytometry measurements of p53 or pRb protein content and DNA content. The hypoxia-induced increase in p53 protein was higher in S-phase than in $G_1$ and $G_2$ phases in all cell lines. The accumulation of p53 in S-phase during hypoxia was not related to hypoxia-induced apoptosis or substantial cell cycle specific cell inactivation during the first 24 h of reoxygenation. pRb was hypophosphorylated in all cell cycle phases by hypoxia treatment. The results did not support a direct link between p53 and pRb during hypoxia because p53 was induced in a cell cycle-specific manner, whereas no cell cycle-dependent differences in pRb hypophosphorylation were detected. Only a fraction of the cell populations (0.60 ± 0.10) showed hypophosphorylated pRb. Thus, pRb is probably not the only mediator of the hypoxia-induced cell cycle block seen in all cells and all cell cycle phases. Moreover, the cell cycle-dependent induction of p53 by hypoxia suggests that the primary function of p53 accumulation during hypoxia is other than to arrest the cells.

**Keywords:** hypoxia; p53; pRb; cell cycle phase

Hypoxia is a common feature of many human tumours (Moulder and Rockwell, 1987; Vaupel et al, 1989). In vivo and in vitro studies of human tumours and cell lines have shown that hypoxia induces treatment resistance (Overgaard et al, 1986; Gatenby et al, 1988; Luk et al, 1990; Höckel et al, 1991; Sanna and Rofstad, 1994) and increases the genomic instability of tumour cells (Hill, 1990; Rofstad et al, 1996*a*). Cell proliferation is reduced or totally inhibited by hypoxia (Pettersen et al, 1986; Moulder and Rockwell, 1987) and cell viability is reduced (Hall et al, 1966; Bedford and Mitchell, 1974). The reduced cell viability can be due to energy depletion, however, other mechanisms might contribute as well (Shrieve et al, 1983; Rotin et al, 1986; Graeber et al, 1996). Hypoxic conditions modulate the expression of certain genes in tumour cells (Brown and Giaccia, 1994). Among these, the products of the tumour-suppressor genes *p53* and *Rb* (the retinoblastoma gene) are important in the regulation of cell cycle progression and cell viability.

The p53 protein is a 53-kDa nuclear phosphoprotein which plays multiple roles in cells. Under normal conditions, little p53 protein is found in most cells. However, when cells are exposed to stimuli that induce DNA damage, the p53 protein is stabilized and accumulates in the nucleus. The p53 protein can, thereafter, act on its transcriptional targets, including the *p21* gene, the *gadd45* gene and the *mdm-2* gene, and arrest the cells in the $G_1$ phase of the cell cycle (Ko and Prives, 1996). It has been suggested that this $G_1$ arrest allows the cells to repair DNA lesions and, thereby, maintain genomic integrity (Lane, 1992). If the repair fails, the accumulation of p53 induced by DNA damage may trigger apoptosis. Hypoxia has been shown to induce accumulation of p53 protein in the nucleus of most cell lines that are wild type for *p53*, but not in cell lines that possess mutant *p53* (Graeber et al, 1994; Åmellem et al, 1997). Cells are arrested in the cell cycle by hypoxia treatment (Graeber et al, 1994; Åmellem and Pettersen, 1991; Ludlow et al, 1993). However, this arrest seems to be independent of the *p53* status (Graeber et al, 1994), and might, therefore, not be caused by the p53 accumulation. Hypoxia treatment can induce apoptosis in some cell lines (Muschel et al, 1995; Yao et al, 1995; Graeber et al, 1996; Shimizu et al, 1996; Åmellem et al, 1997). The role of p53 in hypoxia-induced apoptosis is not completely clarified (Åmellem et al, 1997).

The retinoblastoma protein, pRb, is a nuclear phosphoprotein which regulates the cell cycle. pRb is expressed in all cell types and exists in an active hypophosphorylated and an inactive hyperphosphorylated state (Weinberg, 1995). Under normal conditions, pRb is hypophosphorylated and bound to the nucleus only in early $G_1$ phase (Mittnacht and Weinberg, 1991; Templeton, 1992; Stokke et al, 1993). It is proposed that pRb serves as a brake on the progression of cells from $G_1$ to S-phase of the cell cycle when the protein is in its active state. Hypoxia treatment has been shown to induce reversible hypophosphorylation of pRb in all phases of the cell cycle (Ludlow et al, 1993; Åmellem et al, 1996). This hypoxia-induced hypophosphorylation is probably independent of *p53* status. Moreover, pRb hypophosphorylation does not seem to cause the hypoxia-induced arrest in $G_1$ (Åmellem et al, 1996).

Studies of the cell cycle dependence of hypoxia-induced p53 accumulation have not been reported, nor have measurements of hypoxia-induced changes in p53 and pRb in the same cell line.

*Received 28 January 1998*
*Revised 11 May 1998*
*Accepted 13 May 1998*

*Correspondence to:* T Danielsen

However, knowledge from such studies might elucidate the roles of pRb and p53 in the regulation of cell proliferation and cell viability under hypoxic conditions. In the present work, hypoxia-induced cell cycle-dependent changes in pRb and p53 were investigated in the human melanoma cell lines A-07, D-12, R-18 and U-25 by two-parameter flow cytometry measurements. The cell cycle variability of these gene products was, thereafter, related to proliferation and viability data to reveal information about the functions of p53 and pRb during hypoxia.

### MATERIALS AND METHODS

### Cell lines

Four established human melanoma cell lines (A-07, D-12, R-18, U-25) were used (Rofstad, 1994). Mutations in *p53* were analysed by CDGE (constant denaturant gel electrophoresis) with primers covering exon 5–8 (Børresen, 1996), as well as by complete sequencing of the *p53* cDNA using primers covering the open reading frame (Smith-Sørensen et al, 1998). The analysis showed that all four cell lines express wild-type *p53*. Cell lines were maintained in monolayer culture in RPMI-1640 medium (25 mM HEPES and L-glutamine) supplemented with 13% fetal calf serum, 250 mg $l^{-1}$ penicillin and 50 mg $l^{-1}$ streptomycin. Cultures were incubated at 37°C in a humidified atmosphere of 5% carbon dioxide in air and subcultured by trypsinization (0.05% trypsin/0.02% EDTA solution).

### Exposure to hypoxia

Cells from cultures in exponential growth were plated in glass dishes, incubated at 37°C in a humidified atmosphere of 5% carbon dioxide in air for 24 h, and then exposed to hypoxia for 16 h. The culture medium was removed and replaced by fresh medium supplemented with 2.2 g $l^{-1}$ sodium bicarbonate immediately before hypoxia treatment. The glass dishes were placed in air-tight steel chambers during the hypoxia treatment. The steel chambers were flushed with a humidified, highly purified gas mixture consisting of 95% nitrogen and 5% carbon dioxide at a flow rate of 5 l $min^{-1}$. Measurements showed that the concentration of oxygen in the medium was < 10 p.p.m. after 1 h of flushing. The pH in the medium at the end of the exposure was within the range of 7.3–7.5.

### Cell proliferation and survival during hypoxia

Cell proliferation during hypoxia was determined by counting aerobic control cells or hypoxia-treated cells in a haemocytometer and measuring DNA histograms by flow cytometry as described previously (Sanna and Rofstad, 1994). Cell survival was measured by using a colony assay (Rofstad, 1992). Briefly, 1-ml aliquots of cell suspension were seeded in 25-$cm^2$ tissue culture flasks, containing $10^5$ lethally irradiated feeder cells in 4 ml of medium. After 8–21 days of incubation at 37°C in a humidified atmosphere of 5% carbon dioxide in air, cells were fixed in ethanol, stained with methylene blue, and colonies containing more than 50 cells were counted.

### Apoptosis measurements

Apoptotic cells were detected by immunofluorescence using the ApopTag (Oncor) in situ assay (Yao et al, 1995). Cells washed in $Ca^{2+}$- and $Mg^{2+}$-free Hank's buffered salt solution (HBSS) were resuspended in 4% neutral-buffered paraformaldehyde for 10 min at room temperature. Aliquots of approximately 50 µl of cell suspension were dropped on microscope slides. Two drops of equilibration buffer were added before the slides were incubated at room temperature in a humidified atmosphere for 5 min. Approximately 50 µl of a terminal deoxynucleotide transferase and digoxigenin-11-deoxyuridine triphosphate solution was applied to the cell preparations to end-label DNA fragments. The slides were continuously incubated at 37°C for 1 h and washed in prewarmed stop/wash buffer at 37°C for 30 min. Approximately 50 µl of an antidigoxigenin–fluorescein solution was pipetted onto the slides for detection of the end-labelled DNA fragments before the slides were incubated at room temperature for 30 min. Finally, the slides were mounted under a glass coverslip with a drop of propidium iodide/Antifade staining buffer. The cell preparations were washed three times in phosphate-buffered saline (PBS) between each step in the procedure. The cells were visualized by epifluorescence using standard fluorescein excitation and emission filters. The fraction of cells in apoptosis was determined by scoring 500 cells in each sample.

### Energy charge measurements

The relative concentrations of adenylate phosphates in control cells and cells exposed to hypoxia were measured by high performance liquid chromatography at 254 nm. The medium of the cell cultures was replaced with acetonitrile and the lysed cells were scraped off the glass dishes. The cell lysates were dried and low-strength buffer (100 mM potassium dihydrogen phosphate, 1.5% acetonitrile, 0.08% $C_{16}H_{36}NBr$, pH 5.0) was added before the samples were centrifuged. The supernatant was used in the further analysis. The elution conditions that provided the best resolution were 80% low-strength buffer and 20% high-strength buffer (150 mM potassium dihydrogen phosphate, 10% acetonitrile, 0.08% tetrabutylammonium bromide, pH 5.0) for 10 min, followed by a linear gradient to 100% high-strength buffer during the next 10 min. Elution with the high-strength buffer was continued for another 10 min. The separation was carried out with a Supelcosil LC-18-T 5-µm cartridge (Supelco, USA). The flow rate was 1.0 ml $min^{-1}$. The adenylate energy charge was calculated by the equation:

$$\text{Adenylate energy charge} = \frac{[\text{ATP}] + 1/2[\text{ADP}]}{[\text{ATP}] + [\text{ADP}] + [\text{AMP}]}$$

### Staining for p53 or bound pRb and DNA content

Hypoxia-treated cells were stained for protein and DNA content immediately after exposure to hypoxia. All steps were performed at 0°C. Harvested cells (approximately 2–3 × $10^6$ cells per sample) were washed once in PBS and resuspended in 750 µl of 10 mM phosphate buffer (pH 7.4) containing 0.1% Nonidet P-40, 10 mM sodium chloride, 5 mM magnesium chloride and 0.1 mM phenyl methyl sulphonyl fluoride (PMSF). After 10 min, the extracted cells, which will be termed 'nuclei', were vortexed and 250 µl of 4% paraformaldehyde was added. The nuclei were fixed for 1 h, washed once with staining buffer [150 mM sodium chloride, 0.1% Triton X-100, 2 mM magnesium chloride and 10 mM Tris-HCl (pH 7.4)] and centrifuged. Staining was accomplished by a three-layer procedure. The nuclei were resuspended in 30 µl staining buffer

© Cancer Research Campaign 1998

**Figure 1** The relationship between time of hypoxia treatment and (**A**) the relative cell number of the cell line D-12 at aerobic conditions (●) and hypoxic conditions (■). (**B**) The fraction of D-12 cells in $G_1$ phase (○), S-phase (□) and $G_2$/M phase (△). (**C**) The surviving fraction of D-12 cells after hypoxia treatment. Symbols and bars represent means ± standard errors of five independent experiments containing 5% dry milk. After 10 min, the monoclonal antibody (PAb 1801, p53 Ab-2, Oncogene Science; PMG3-245, pRb, Pharmingen) diluted in staining buffer was added to a final concentration of 2.0 μg $ml^{-1}$ (p53) or 10.0 μg $ml^{-1}$ (pRb) in a total volume of 60 μl. The control samples received no primary antibody. After incubation with the first layer for 1 h, the nuclei were washed once with staining buffer and resuspended in 60 μl secondary antibody (biotinylated anti-mouse Ig from sheep, Amersham Life Science) diluted 1:50 in staining buffer. The nuclei were incubated for 30 min, washed with staining buffer and resuspended in 60 μl streptavidin–FITC (FITC, fluorescein isothiocyanate) (Amersham Life Science) diluted 1:50 in staining buffer. After incubation for 30 min, the nuclei were washed once with staining buffer and resuspended in 500 μl of staining buffer containing 2 μg $ml^{-1}$ Hoechst 33258. The samples were filtered through a 30-μm nylon mesh and analysed in a flow cytometer.

### Staining for BrdUrd and DNA content

To investigate the progression of cells in the different phases of the cell cycle after hypoxia treatment, cells were incubated with 50 μg $ml^{-1}$ BrdUrd (5-bromo-2′-deoxyuridine) for 30 min immediately before hypoxia treatment. The culture medium containing BrdUrd was removed, the cells were washed several times with PBS and fresh medium supplemented with sodium bicarbonate was added before hypoxia treatment. The cells were incubated at 37°C in a humidified atmosphere of 5% carbon dioxide in air for various times after reoxygenation. Cells were harvested and fixed in 100% methanol and stored at −20°C. Immunostaining for BrdUrd was performed according to the protocol of Gerlyng et al (1992). Briefly, the cells were digested in a suspension of 0.2% pepsin in 2 M hydrochloric acid and stained for BrdUrd by a three-layer procedure. The anti-BrdUrd antibody (Becton Dickinson), the secondary antibody (biotinylated anti-mouse Ig from sheep, Amersham Life Science) and streptavidin–FITC (Amersham Life Science) were diluted in PBS containing 0.5% Tween 20 and 0.5% bovine serum albumin. PBS was employed for washing between incubations. After incubation with streptavidin–FITC, the pellets were washed once with PBS and resuspended in 500 μl of PBS with 2.5 μg $ml^{-1}$ propidium iodide (Calbiochem) and 100 μg $ml^{-1}$ RNAase A (Pharmacia). The samples were filtered through a 30-μm nylon mesh and analysed in a flow cytometer.

### Flow cytometry

The stained cells or nuclei were analysed in a FACStar$^{PLUS}$ flow cytometer (Becton Dickinson) equipped with two argon lasers (SpectraPhysics) that were tuned to 488 nm and UV respectively. FITC fluorescence, forward light scatter and side scatter pulse amplitudes, as well as propidium iodide fluorescence pulse height, pulse width and pulse area were measured upon excitation by the 488-nm laser. Hoechst 33258 fluorescence pulse height, pulse width and pulse area were measured upon excitation by the UV laser. Hoechst 33258 fluorescence pulse area or propidium iodide fluorescence pulse area were used as a measure of DNA content. The nuclei were gated for doublet discrimination in a diagram of integrated Hoechst 33258 fluorescence against Hoechst 33258 pulse width. The green fluorescence intensities were calibrated with fluorescent beads in each experiment, such that the FITC fluorescence intensities measured in different experiments could be compared.

### Data treatment and analysis

The median FITC fluorescence intensity (FL1-H) of the anti-p53-stained sample subtracted by the median fluorescence intensity of the corresponding control sample (which had received no primary antibody) was used as a measure of relative level of p53 protein in the nuclei. Measurements of relative levels of p53 protein in each phase of the cell cycle were obtained by generating FL1-H histograms from three narrow gates in the DNA histograms. T-47D

© Cancer Research Campaign 1998

**Figure 2** Flow cytometric analysis of D-12 nuclei stained for p53 protein and DNA content. The histograms show DNA content (integrated Hoechst 33258 fluorescence; F32-A) and p53 protein content (FITC fluorescence; FL1-H). Histograms A–D represent aerobic cells, whereas histograms E–K represent cells exposed to hypoxia for 16 h. The dual parameter histograms A and E represent control cells which received no primary antibody against p53. The dual parameter histograms B and F show the distribution of stained p53 protein in the nuclei throughout the cell cycle. The DNA content of these nuclei is also shown in one-parameter F32-A histograms (C and G). The gates R1, R2 and R3 in these histograms were applied to generate FL1-H histograms for cells in $G_1$ phase, S-phase and $G_2$ phase of the cell cycle respectively. p53 protein content of different cell cycle phases is shown for hypoxia-treated cells in histograms I–K. Histograms for total p53 protein level are shown in D and H

cells, which are mutant for *p53*, were used as a positive control. They showed a relative level of p53 protein that was about 20 times the value of the untreated melanoma cells, which are wild type for *p53*.

The extraction procedure provides samples where only the detergent-resistant bound pRb is present, i.e. hypophosphorylated pRb (Mittnacht and Weinberg, 1991; Templeton, 1992; Stokke et al, 1993). At normal conditions, pRb is only bound in the nucleus in $G_1$. Two distinct peaks with $G_1$ DNA content were observed in the FL1-H histograms (FITC fluorescence) of aerobic nuclei. Previous studies have shown that the PMG3-245 anti-pRb monoclonal antibody has a high degree of non-specific binding (Stokke et al, 1993; Jonassen et al, 1994). The nuclei with low FL1-H fluorescence intensities were, therefore, assumed to have only non-specific binding of the pRb antibody, and were termed pRb$^-$ nuclei. High FL1-H fluorescence intensities were assumed to

© Cancer Research Campaign 1998

**Figure 3** Calculated values of relative levels of p53 protein in the nuclei of aerobic cells (●) and hypoxic cells (○) in each phase of the cell cycle. Five independent experiments with aerobic cells and cells treated with hypoxia for 16 h were carried out for the four cell lines (**A**) A-07, (**B**) D-12, (**C**) R-18 and (**D**) U-25

**Figure 4** Western blot showing the expression of p53 in A-07, D-12, R-18 and U-25 cells from aerobic control cells (C) and hypoxia-treated cells (H). Proteins from 5 × $10^5$ cells were loaded in each lane. The band below p53 was due to non-specific binding, as shown in blots stained without the p53 antibody represent nuclei with bound hypophosphorylated pRb, which were called $pRb^+$ nuclei. The fraction of $pRb^+$ nuclei was obtained by measuring the relative number of nuclei with high FL1-H. In addition to omission of the primary antibody, the B-cell lymphoma cell line U698 was used as a negative control.

### Western blotting

Cells harvested from cultures in exponential growth were boiled in Laemmli's lysis buffer (Laemmli, 1970) for 5 min. Proteins were separated by 9% sodium dodecyl sulphate polyacrylamide gel electrophoresis and transferred to a polyvinylidene fluoride (PVDF) membrane. The mouse monoclonal antibodies PAb1801 (Oncogene Science) and PMG3-245 (Pharmingen) were used for specific staining of p53 and pRb. Staining was performed using a three-layer procedure, using biotinylated anti-mouse antibody (Amersham Life Science) as second layer, streptavidin–alkaline phosphatase (Amersham Life Science) as third layer and BCIP/NBT (Sigma) as substrate.

### Statistical analysis

Statistical comparisons of data were performed by parametric analysis using Student's *t*-tests and one-way analysis of variance. Multivariate statistical analysis was applied to compare measured quantities in the phases of the cell cycle for each of the four cell lines (Johnsen and Wichern, 1992). A significance criterion of $P < 0.05$ was used.

### RESULTS

### The melanoma cells are arrested in all cell cycle phases during hypoxia treatment

The cell number of the D-12 line remained constant during hypoxia treatment (Figure 1A). Measurements of the cell cycle distribution of D-12 cells by flow cytometry revealed no significant changes during hypoxia treatment (Figure 1B). Similar results were obtained for the other three lines. The constant cell number together with the unchanged cell cycle distribution during hypoxia treatment indicate that the melanoma cells were growth arrested in all phases of the cell cycle immediately after the hypoxic environment was established.

### Hypoxia treatment does not induce apoptosis, although the energy status of the melanoma cells is slightly decreased

The survival of D-12 cells decreased with increasing time of hypoxia treatment (Figure 1C), with a mean surviving fraction of 0.45 after 16 h of hypoxia treatment. Quantitatively similar survival curves were obtained for the other three melanoma lines. The fraction of cells that were scored apoptotic was less than 2% after 16 h of hypoxia treatment. This fraction was not significantly different from that of aerobic control cells ($P > 0.05$) for any of the melanoma lines (data not shown). The adenylate energy charge values (mean ± s.e.) after 16 h of hypoxia treatment were 0.79 ± 0.03 (A-07), 0.84 ± 0.03 (D-12), 0.84 ± 0.02 (R-18) and 0.68 ± 0.02 (U-25). The control values for aerobic cells were 0.92 ± 0.01 (A-07), 0.94 ± 0.01 (D-12) and 0.91 ± 0.01 (R-18 and U-25). The adenylate energy charge was significantly lower for the hypoxia-treated cells than for the control cells for all cell lines ($P < 0.0001$).

### p53 protein is induced specifically in S-phase under hypoxic conditions

An example of results from flow cytometric analysis of aerobic control cells and hypoxia-treated D-12 cells which were stained for p53 protein and DNA content is shown in Figure 2. The histograms in Figure 2B and F show the distributions of p53 protein throughout the cell cycle for aerobic control cells and hypoxia-treated cells respectively. Hypoxic conditions induced accumulation of p53 protein in a cell cycle-dependent manner. To investigate this cell cycle-dependent accumulation of p53 protein more precisely, histograms of p53 protein content were generated for each phase of the cell cycle using narrow gates set in the DNA histograms (Figure 2C and G). Figure 2I–K shows histograms of p53 protein content in hypoxia-treated cells in $G_1$ phase, S-phase and $G_2$ phase respectively. Median values of histograms like these were used to calculate the relative levels of p53 protein in the different cell cycle phases.

© Cancer Research Campaign 1998 

**Figure 5** Flow cytometric analysis of D-12 nuclei stained for pRb protein and DNA content. The histograms show DNA content (integrated Hoechst 33258 fluorescence: F32-A) and pRb protein content (FITC fluorescence: FL1-H). Histograms **A–F** represent aerobic cells, whereas histograms **G–L** represent cells exposed to hypoxia for 16 h. (**A** and **G**) Gate R4 includes cells that are pRb⁻ and gate R5 includes cells that are pRb⁺. For the total cell population, the fraction of pRb⁻ nuclei was set equal to the relative number of nuclei within the region limited by gate R5. The gates R1, R2 and R3 set in the DNA histograms in **B** and **H** were used to generate FL1-H histograms for each phase of the cell cycle. (**D** and **J**) FL1-H histograms for nuclei in $G_1$ phase. (**E** and **K**) FL1-H histograms for S-phase nuclei. (**F** and **L**) FL1-H histograms for nuclei in $G_2$ phase. The fractions of pRb⁻ nuclei in the phases of the cell cycle were obtained by measuring the relative number of nuclei in the peak with high FL1-H fluorescence intensity in these six histograms. (**C** and **I**) FL1-H histograms for the total cell population. Control samples which were not treated with primary antibody are not shown, however, they were similar to the control samples for p53 protein staining shown in Figure 3A and E

 © Cancer Research Campaign 1998

**Figure 6** Fraction $pRb^-$ nuclei in aerobic cells (●) and hypoxia-treated cells (○) through the cell cycle for the cell lines (**A**) A-07, (**B**) D-12, (**C**) R-18 and (**D**) U-25. Five independent experiments with aerobic cells and cells treated with hypoxia for 16 h were carried out

Measurements of relative levels of p53 protein in each phase of the cell cycle under aerobic and hypoxic conditions are shown in Figure 3. The relative level of p53 protein tended to increase in all cell cycle phases after hypoxia treatment. However, the hypoxia-induced accumulation of p53 protein in S-phase was the dominating effect, and a significant increase in p53 protein was seen in all cell lines (A-07, D-12, R-18, $P < 0.0005$; U-25, $P = 0.01$). Moreover, the increase in p53 protein in S-phase was significantly higher than in $G_1$ phase in all cell lines ($P < 0.05$), and significantly higher than in $G_2$ phase in all cell lines ($P < 0.05$) except in R-18 ($P = 0.054$). The total cell populations (Figure 2D and H) also showed increases in p53 protein under hypoxic conditions that were significant for all the cell lines (A-07, $P = 0.01$; D-12, $P = 0.0009$; R-18, $P = 0.03$; U-25, P = 0.002).

Western blotting of whole cell lysates showed p53 induction after hypoxia treatment, in agreement with the flow cytometry results (Figure 4).

### The retinoblastoma protein is hypophosphorylated and bound in the nucleus in all phases of the cell cycle under hypoxic conditions

Figure 5 shows an example of results from flow cytometric analysis of aerobic (A–F) and hypoxia-treated (G–L) D-12 cells which were stained for retinoblastoma protein and DNA content. Under aerobic conditions, all the cell lines showed $pRb^-$ nuclei in the $G_1$ phase (Figure 5A). Figure 5G illustrates that hypoxia treatment induced pRb bound in the nucleus in all phases of the cell cycle. Similar histograms were obtained for the other cell lines. Histograms of pRb content in each phase of the cell cycle were generated by the same method as for p53.

Measured fractions of $pRb^-$ nuclei in the phases of the cell cycle for untreated and hypoxia-treated cells are shown in Figure 6. The fraction of $pRb^-$ nuclei in the $G_1$ phase of aerobic cells did not differ significantly between the four cell lines. The cell lines had a

© Cancer Research Campaign 1998

**Figure 7** Western blot showing the expression of pRb in A-07, D-12, R-18 and U-25 cells from aerobic control cells (C) and hypoxia-treated cells (H). Proteins from $5 \times 10^5$ cells were loaded in each lane. The band above pRb was due to non-specific binding, as shown in blots stained without the pRb antibody fraction of ($0.18 \pm 0.05$) $pRb^+$ nuclei in the $G_1$ phase of the cell cycle under aerobic conditions. The increase in fraction of $pRb^+$ nuclei after hypoxia treatment was statistically significant ($P < 0.05$) in any phase of the cell cycle in all cell lines. For hypoxia-treated cells, the fraction of $pRb^+$ nuclei did not vary significantly throughout the cell cycle. The total cell populations were, therefore, used when comparing the cell lines. Comparison of the fractions of $pRb^+$ nuclei of hypoxia-treated cells resulted in no statistically significant differences between the cell lines ($P > 0.05$). The fraction of the nuclei that were $pRb^+$ under hypoxic conditions was $0.60 \pm 0.10$.

Western blotting of whole cell lysates showed accumulation of hypophosphorylated pRb after hypoxia treatment (Figure 7), consistent with the observation of pRb binding to the nucleus after hypoxia seen in the flow cytometry experiments.

### BrdUrd analysis does not reveal substantial hypoxia-induced cell cycle phase-specific cell inactivation

Some studies have shown that cells in S-phase at the start of hypoxia treatment are most sensitive to the lethal effects of hypoxia (Spiro et al, 1984; Åmellem and Pettersen, 1991). On the basis of the present observations of S-phase-specific p53 induction by hypoxia, experiments were performed to investigate whether hypoxia treatment induced phase-specific cell inactivation, and particularly whether cells in S-phase were more sensitive to hypoxia treatment than cells in other phases. Cells were labelled with BrdUrd for 30 min before hypoxia treatment to follow the cell cycle distribution after reoxygenation. Cells that are synthesizing DNA (S-phase cells) during the labelling period will incorporate BrdUrd and show a high FITC fluorescence compared with cells that are not synthesizing DNA ($G_1$ and $G_2/M$ phase cells). The hypoxia-treated cells were reoxygenated and fixed at several time points after reoxygenation. Cells that are reoxygenated after hypoxia treatment will re-enter the cell cycle because they are metabolically viable immediately after hypoxia treatment (Kwok and Sutherland, 1989). However, a certain number of the cells are clonogenically dead, and will at some time point after reoxygenation stop cycling and disintegrate. These dead cells will not attach to the surface of the glass dishes, and will, therefore, not be a part of the fixed cell population. Thus, analysis of BrdUrd versus DNA content over a broad time period after reoxygenation should reveal whether the majority of the inactivated cells were in S-phase during hypoxia.

An example of results from flow cytometric analysis of aerobic control cells and hypoxia-treated A-07 cells stained for BrdUrd and DNA content is shown in Figure 8. The distribution of BrdUrd-negative (R1) and BrdUrd-positive (R2) aerobic control cells through the cell cycle immediately after BrdUrd incorporation is shown in Figure 8A. The cell cycle distribution of BrdUrd-negative (R1) and BrdUrd-positive (R2) hypoxia-treated cells is shown at 0 h (B), 4 h (C) and 13 h (D) after hypoxia treatment. As expected from Figure 1B, there were no significant differences between the aerobic control cells (Figure 8A) and the hypoxia-treated cells (Figure 8B) immediately after BrdUrd incorporation or hypoxia treatment. With increasing reoxygenation times (up to 24 h), the BrdUrd-positive hypoxia-treated cells became gradually distributed throughout all phases of the cell cycle. Four hours after reoxygenation, BrdUrd-labelled cells (R2) from hypoxia-treated populations had moved on to $G_2/M$ and the next $G_1$ (Figure 8C), although the cell cycle time of the hypoxia-treated cells seemed to be increased compared with aerobic control cells (data not shown). Thirteen hours after reoxygenation (Figure 8D), cells that were in S-phase during hypoxia were distributed among all cell cycle phases. Thus, at least some of the cells which were in S-phase during the hypoxia treatment were still cycling. Cells in $G_1$ and $G_2/M$ phase during hypoxia treatment (BrdUrd-negative cells) were also cycling (Figure 8D). Moreover, the ratio of BrdUrd-negative and BrdUrd-positive hypoxia-treated cells did not vary substantially during the first 24 h of reoxygenation. The ratios of BrdUrd-negative and BrdUrd-positive cells at time points later than 24 h were not analysed, as they were disturbed by cell division. Thus, the BrdUrd analysis did not reveal any substantial hypoxia-induced cell cycle-specific inactivation during the first 24 h of reoxygenation.

### DISCUSSION

Cells expressing wild-type *p53* accumulate p53 protein when exposed to stresses such as ionizing radiation, UV light, heat shock, starvation and hypoxia (Ko and Prives, 1996). We found a significant hypoxia-induced increase in p53 protein level in the S-phase of four melanoma cell lines that are wild type for *p53*. Moreover, the increase in p53 level in S-phase was significantly higher than in $G_1$ phase in all cell lines, and significantly higher than in $G_2$ phase in three out of four cell lines. Cell cycle-dependent accumulation of p53 by hypoxia treatment has not been reported previously. In a recent study of three human wild-type *p53* cell lines, γ-irradiation was shown to induce p53 selectively in $G_1$ phase and early S-phase (Komarova et al, 1997). Moreover, UV radiation of wild-type *p53* NIH3T3 cells induced p53 selectively in the S-phase of cells that were synchronized by serum starvation (Haapajärvi et al, 1995). In contrast, p53 induction by heat shock and UV has been shown to be independent of cell cycle phase in human fibroblasts (Yamaizumi and Sugano, 1994; Sugano et al, 1995). The p53 induction by hypoxia, however, cannot be compared with that of DNA-damaging treatments because hypoxia probably induces little or no DNA damage. Whereas DNA lesions in cells may trigger p53 accumulation to maintain genomic integrity (Lane, 1992), neither the trigger for hypoxia-induced p53 accumulation nor all cellular effects of p53 during hypoxia are known.

In the present study, p53 induction by hypoxia was only studied in melanoma cell lines that are wild type for *p53*. There are rather few reports in which cell lines with mutations in the *p53* gene have been exposed to hypoxia. Graeber et al (1994) reported no increase in nuclear p53 protein levels of human prostate cell lines DU-145 and PC-3, which contain only mutant *p53*, after exposure to

 © Cancer Research Campaign 1998

**Figure 8** Flow cytometric analysis of A-07 cells stained for BrdUrd and DNA content. The cells were labelled with BrdUrd for 30 min before hypoxia treatment. The histograms show DNA content (integrated propidium iodide fluorescence: F31-A) and BrdUrd content (FITC fluorescence: FL1-H). **A** represents aerobic control cells, whereas **B–D** represent cells exposed to hypoxia for 16 h. Cells were fixed at 0 h (**A**, **B**), 4 h **C**), or 13 h (**D**) after BrdUrd incorporation (aerobic cells) or hypoxia treatment. In the dual parameter histograms, BrdUrd-negative (R1) and BrdUrd-positive (R2) cells are gated. The fraction of cells (in per cent) that are BrdUrd-negative and BrdUrd-positive is shown above the gated areas. The DNA content of BrdUrd-negative and BrdUrd-positive cells is shown to the right of the dual parameter histograms hypoxia. Moreover, no induction of p53 protein was observed when the mutant *p53*-expressing breast cancer cell line T47-D was treated with hypoxia (Åmellem et al, 1997). These observations are in agreement with the lack of increase in p53 levels seen in most cells with mutant *p53* when exposed to DNA-damaging agents (Kastan et al, 1991; O'Connor et al, 1993).

Hypoxia treatment induced accumulation of hypophosphorylated pRb in all the melanoma cell lines. However, only a fraction of the cells showed hypophosphorylated pRb bound to the nucleus. This cell fraction did not seem to differ between the cell cycle phases. Our observations are consistent with those of Åmellem et al (1996), who found that pRb was hypophosphorylated in only a certain fraction of the cell populations when the cells were exposed to less than 4 p.p.m. oxygen. In their work, however, the fraction of the cells with hypophosphorylated pRb differed between cell lines, whereas no significant differences could be detected between the four melanoma lines studied here.

p53 accumulation induced by DNA-damaging agents can mediate $G_1$ phase arrest (Ko and Prives, 1996). $G_1$ arrest by hypoxia, however, seems to be independent of p53 accumulation (Graeber et al, 1994). The arrest of the melanoma cells in all phases of the cell cycle did not correlate with the cell cycle-dependent induction of p53. The primary function of p53 induction by hypoxia treatment is, therefore, probably not to arrest the cells. Consequently, we suggest that factors other than p53 are essential for the hypoxia-induced cell cycle arrest of the melanoma cells. Several physiological growth inhibitory signals as well as DNA-damaging agents can block pRb phosphorylation and, thereby, arrest the cells in $G_1$ phase (Weinberg, 1995). However, because only a fraction of the melanoma cells showed pRb hypophosphorylation by hypoxia, pRb activation is probably not the only cause of the hypoxia-induced cell cycle arrest as all cells were arrested. Thus, pRb hypophosphorylation does not seem to be essential for the hypoxia-induced cell cycle arrest of the melanoma cells, in accordance with the observations of Åmellem et al (1996).

The arrest of cells in the cycle has been observed in different cell lines when exposed to a sufficiently low oxygen concentration. However, although all cell lines are arrested on the $G_1$/S border by hypoxia, some cell lines progress from mitosis to $G_1$ (Spiro et al, 1984; Åmellem and Pettersen, 1991), whereas others are also arrested in mitosis (Shrieve et al, 1983, the present study). The differences in cell cycle arrest between cell lines are probably not caused by different levels of oxygenation, as all investigators used an oxygen concentration of less than 10 p.p.m. Thus, there seems to be cell line-specific differences in the regulation of the hypoxia-induced cell cycle arrest. As most of the cell lines used in these experiments are tumour lines, such differences could be due to mutations, causing changes in the expression of so far unknown proteins regulating the cell cycle progression during hypoxia.

Several studies have suggested a link between p53 and pRb in the control of cell growth (Demers et al, 1994; Hickman et al, 1994; Slebos et al, 1994; Haupt et al, 1995). When cells are exposed to DNA-damaging agents, the p53 induction leads to an increase in p21. At high protein concentrations, p21 inhibits the functions of CDKs (cyclin-dependent kinases), allowing the accumulation of hypophosphorylated pRb, which might result in radiation-induced $G_1$ arrest. Hypoxia, however, has not been shown to activate this $G_1$ block pathway. The present data do not support a link between p53 and pRb during hypoxia because p53 was induced in a cell cycle-specific manner, whereas no cell cycle-dependent differences in the fraction of cells with hypophosphorylated pRb were detected. Our observations are consistent with those of Graeber and co-workers (Graeber et al, 1994), who suggested that hypoxia induces p53 protein by a different pathway to DNA damaging agents. Moreover, hypoxia-induced hypophosphorylation of pRb is probably not dependent on functional *p53* (Åmellem et al, 1996).

Hypoxic conditions of 10 p.p.m. oxygen for some hours inevitably led to cell inactivation, despite the ability of cells to adapt to an oxygen-poor atmosphere. In the melanoma lines, approximately one-half of the cells lost clonogenicity after 16 h of hypoxia treatment. This cell inactivation is probably not due to energy depletion, as adenylate energy charge values after 16 h of hypoxia treatment were rather high [between 0.68 ± 0.02 (U-25) and 0.84 ± 0.03 (D-12 and R-18)]. Because the adenylate energy charge of most normal cells are in the range 0.80–0.95 (Stryer, 1988), it is unlikely that the hypoxia-induced decrease in energy charge will lead to substantial cell inactivation. Results from studies of rodent cell lines support the present observation, as they suggest that other mechanisms were involved in the hypoxia-induced cell inactivation (Shrieve et al, 1983; Rotin et al, 1986). However, the mechanism by which hypoxia induces cell inactivation has not been fully elucidated. In the present work, we attempted to clarify the roles of p53 and pRb in hypoxia-induced cell inactivation.

Induction of apoptosis has been shown to contribute to hypoxia-induced cell death (Muschel et al, 1995; Yao et al, 1995; Graeber et al, 1996; Rofstad et al, 1996*b*; Shimizu et al, 1996). Furthermore, a relationship between the accumulation of p53 during hypoxia and hypoxia-induced apoptosis has been suggested (Graeber et al, 1996), as a parallel to the p53-dependent apoptotic pathway induced by DNA damage. The hypoxia-induced p53 accumulation in S-phase melanoma cells did not induce apoptosis. Whereas all the melanoma lines showed a significant increase in p53 level after 16 h of hypoxia treatment, none of the melanoma lines showed a significant increase in the apoptotic fraction. The lack of apoptosis induction despite the significant p53 accumulation in the melanoma lines suggests that induction of p53 by hypoxia does not necessarily lead to apoptosis in tumour cells that are wild type for *p53*. A possible overexpression of the proteins Bcl-2 and Bcl-$x_L$ might explain the lack of hypoxia-induced apoptosis in the melanoma cell lines, as these proteins have been shown to prevent hypoxia-induced apoptosis (Graeber et al, 1996; Shimizu et al, 1996).

Some studies have shown, by measuring clonogenic survival, that cells in S-phase at the start of hypoxia treatment are most sensitive to the lethal effects of hypoxia (Spiro et al, 1984; Åmellem and Pettersen, 1991). However, others have found little difference in the survival of cells to hypoxia during the cell cycle (Kwok and Sutherland, 1989). The BrdUrd analysis of melanoma cells did not reveal any substantial cell cycle phase-specific inactivation during the first 24 h after hypoxia treatment. However, as the applied method is rather approximate, we cannot exclude the possibility that a larger fraction of S-phase cells than of $G_1$ and $G_2$/M cells are inactivated after hypoxia treatment. Nevertheless, our results suggest that many cells residing in S-phase during hypoxia treatment survive, in contrast to a previous study on human cells (Åmellem and Pettersen, 1991).

In conclusion, four human melanoma cell lines exposed to hypoxia showed accumulation of p53 primarily in the S-phase and induction of hypophosphorylated pRb in all phases of the cell cycle. The hypoxia-induced p53 accumulation was not associated with apoptosis or cell cycle-specific cell inactivation. Moreover,

© Cancer Research Campaign 1998

neither p53 accumulation nor pRb hypophosphorylation seemed to be essential for the hypoxia-induced cell cycle block seen in all phases of the cell cycle.

### ACKNOWLEDGEMENTS

We thank professor Anne-Lise Børresen-Dale and Birgitte Smith-Sørensen, PhD, at the Department of Genetics, The Norwegian Radium Hospital, for the *p53* mutation analyses. Financial support was received from The Norwegian Cancer Society.

### REFERENCES

Åmellem Ø and Pettersen EO (1991) Cell inactivation and cell cycle inhibition as induced by extreme hypoxia: the possible role of cell cycle arrest as a protection against hypoxia-induced lethal damage. *Cell Proliferation* **24**: 127–141

Åmellem Ø, Stokke T, Sandvik JA and Pettersen EO (1996) The retinoblastoma gene product is reversibly dephosphorylated and bound in the nucleus in S and G2 phases during hypoxic stress. *Exp Cell Res* **227**: 106–115

Åmellem Ø, Stokke T, Sandvik JA, Smedshammer L and Pettersen EO (1997) Hypoxia-induced apoptosis in human cells with normal p53 status and function, without any alteration in the nuclear protein level. *Exp Cell Res* **232**: 361–370

Bedford JS and Mitchell JB (1974) The effect of hypoxia on the growth and radiation response of mammalian cells in culture. *Br J Radiol* **47**: 687–696

Brown JM and Giaccia AJ (1994) Tumour hypoxia: the picture has changed in the 1990s. *Int J Radiat Biol* **65**: 95–102

Børresen A-L (1996) Constant denaturant gel electrophoresis (CDGE) in mutation screening. In *Technologies for Detection of DNA Damage and Mutation*. Pfeifer GP (ed.), pp. 267–279. Plenum Press: New York

Demers GW, Foster SA, Halbert CL and Galloway DA (1994) Growth arrest by induction of p53 in DNA damaged keratinocytes is bypassed by human papillomavirus 16 E7. *Proc Natl Acad Sci USA* **91**: 4382–4386

Gatenby RA, Kessler HB, Rosenblum JS, Coia LR, Moldofsky PJ, Hartz WH and Broder GJ (1988) Oxygen distribution in squamous cell carcinoma metastases and its relationship to outcome of radiation therapy. *Int J Radiat Oncol Biol Phys* **14**: 831–838

Gerlyng P, Stokke T, Huitfeldt HS, Stenersen T, Danielsen HE, Grotmol T and Seglen PO (1992) Analytical methods for the study of liver cell proliferation. *Cytometry* **13**: 404–415

Graeber TG, Peterson JF, Tsai M, Monica K, Fornace AJJ and Giaccia AJ (1994) Hypoxia induces accumulation of p53 protein, but activation of a G1-phase checkpoint by low-oxygen conditions is independent of p53 status. *Mol Cell Biol* **14**: 6264–6277

Graeber TG, Osmanian C, Jacks T, Housman DE, Koch CJ, Lowe SW and Giaccia AJ (1996) Hypoxia-mediated selection of cells with diminished apoptotic potential in solid tumours. *Nature* **379**: 88–91

Haapajärvi T, Kivinen L, Pitkänen K and Laiho M (1995) Cell cycle dependent effects of u.v.-radiation on p53 expression and retinoblastoma protein phosphorylation. *Oncogene* **11**: 151–159

Hall EJ, Bedford JS and Oliver R (1966) Extreme hypoxia: its effect on the survival of mammalian cells irradiated at high and low dose-rates. *Br J Radiol* **39**: 302–307

Haupt Y, Rowan S and Oren M (1995) p53-mediated apoptosis in HeLa cells can be overcome by excess pRB. *Oncogene* **10**: 1563–1571

Hickman ES, Picksley SM and Vousden KH (1994) Cells expressing HPV16 E7 continue cell cycle progression following DNA damage induced p53 activation. *Oncogene* **9**: 2177–2181

Hill RP (1990) Tumor progression: potential role of unstable genomic changes. *Cancer Metastasis Rev* **9**: 137–147

Höckel M, Schlenger K, Knoop C and Vaupel P (1991) Oxygenation of carcinomas of the uterine cervix: evaluation by computerized $O_2$ tension measurements. *Cancer Res* **51**: 6098–6102

Johnsen RA and Wichern DW (1992) *Applied Multivariate Statistical Analysis*. Prentice Hall International: Englewood Cliffs, New Jersey

Jonassen TS, Seglen PO and Stokke T (1994) The fraction of cells in $G_1$ with bound retinoblastoma protein increases with the duration of the cell cycle. *Cell Proliferation* **27**: 95–104

Kastan MB, Onyekwere O, Sidransky D, Vogelstein B and Craig RW (1991) Participation of p53 protein in the cellular response to DNA damage. *Cancer Res* **51**: 6304–6311

Ko LJ and Prives C (1996) p53: puzzle and paradigm. *Genes Dev* **10**: 1054–1072

Komarova EA, Zelnick CR, Chin D, Zeremski M, Gleiberman AS, Bacus SS and Gudkov AV (1997) Intracellular localization of p53 tumor suppressor protein in gamma-irradiated cells is cell cycle-regulated and determined by the nucleus. *Cancer Res* **57**: 5217–5220

Kwok TT and Sutherland RM (1989) The radiation response of cells recovering after chronic hypoxia. *Radiat Res* **119**: 261–267

Lemmli UK (1970) Cleavage of structural proteins during the assembly of the head of bacteriophage T4. *Nature* **227**: 680–685

Lane DP (1992) p53, guardian of the genome. *Nature* **358**: 15–16

Ludlow JW, Howell RL and Smith HC (1993) Hypoxic stress induce reversible hypophosphorylation of pRB and reduction in cyclin A abundance independent of cell cycle progression. *Oncogene* **8**: 331–339

Luk CK, Veinot-Drebot L, Tjan E and Tannock IF (1990) Effect of transient hypoxia on sensitivity to doxorubicin in human and murine cell lines. *J Natl Cancer Inst* **82**: 684–692

Mittnacht S and Weinberg RA (1991) G1/S phosphorylation of the retinoblastoma protein is associated with an altered affinity for the nuclear compartment. *Cell* **65**: 381–393

Moulder JE and Rockwell S (1987) Tumor hypoxia: its impact on cancer therapy. *Cancer Metastasis Rev* **5**: 313–341

Muschel RJ, Bernhard EJ, Garza L, McKenna WG and Koch CJ (1995) Induction of apoptosis at different oxygen tensions: evidence that oxygen radicals do not mediate apoptotic signalling. *Cancer Res* **55**: 995–998

O'Connor PM, Jackman J, Jondle D, Bhatia K, Magrath I and Kohn KW (1993) Role of the p53 tumor suppressor gene in cell cycle arrest and radiosensitivity of Burkitt's lymphoma cell lines. *Cancer Res* **53**: 4776–4780

Overgaard J, Hansen HS, Jørgensen K and Hjelm HM (1986) Primary radiotherapy of larynx and pharynx carcinoma – an analysis of some factors influencing local control and survival. *Int J Radiat Oncol Biol Phys* **12**: 515–521

Pettersen EO, Juul NO and Rønning ØW (1986) Regulation of protein metabolism of human cells during and after acute hypoxia. *Cancer Res* **46**: 4346–4351

Rofstad EK (1992) Retention of cellular radiation sensitivity in cell and xenograft lines established from human melanoma surgical specimens. *Cancer Res* **52**: 1764–1769

Rofstad EK (1994) Orthotopic human melanoma xenograft model systems for studies of tumour angiogenesis, pathophysiology, treatment sensitivity and metastatic pattern. *Br J Cancer* **70**: 804–812

Rofstad EK, Johnsen NM and Lyng H (1996*a*) Hypoxia-induced tetraploidisation of a diploid human melanoma cell line in vitro. *Br J Cancer* **74**: S136–S139

Rofstad EK, Eide K, Skøyum R, Hystad ME and Lyng H (1996*b*) Apoptosis, energy metabolism, and fraction of radiobiologically hypoxic cells: a study of human melanoma multicellular spheroids. *Int J Radiat Biol* **70**: 241–249

Rotin D, Robinson B and Tannock IF (1986) Influence of hypoxia and an acidic environment on the metabolism and viability of cultured cells: potential implications for cell death in tumors. *Cancer Res* **46**: 2821–2826

Sanna K and Rofstad EK (1994) Hypoxia-induced resistance to doxorubicin and methotrexate in human melanoma cell lines in vitro. *Int J Cancer* **58**: 258–262

Shimizu S, Eguchi Y, Kamiike W, Itoh Y, Hasegawa J, Yamabe K, Otsuki Y, Matsuda H and Tsujimoto Y (1996) Induction of apoptosis as well as necrosis by hypoxia and predominant prevention of apoptosis by Bcl-2 and Bcl-XL. *Cancer Res* **56**: 2161–2166

Shrieve DC, Deen DF and Harris JW (1983) Effects of extreme hypoxia on the growth and viability of EMT6/SF mouse tumor cells in vitro. *Cancer Res* **43**: 3521–3527

Slebos RJ, Lee MH, Plunkett BS, Kessis TD, Williams BO, Jacks T, Hedrick L, Kastan MB and Cho KR (1994) p53-dependent G1 arrest involves pRB-related proteins and is disrupted by the human papillomavirus 16 E7 oncoprotein. *Proc Natl Acad Sci USA* **91**: 5320–5324

Smith-Sørensen B, Kærn J, Holm R, Dørum A, Tropé C and Børresen-Dale A-L (1998) Therapy effect of either paclitaxel or cyclophosphamide combination treatment in patients with epithelial ovarian cancer and relation to *TP53* status. *Br J Cancer* (in press)

Spiro IJ, Rice GC, Durand RE, Stickler R and Ling CC (1984) Cell killing, radiosensitization and cell cycle redistribution induced by chronic hypoxia. *Int J Radiat Oncol Biol Phys* **10**: 1275–1280

Stokke T, Erikstein BK, Smedshammer L, Boye E and Steen HB (1993) The retinoblastoma gene product is bound in the nucleus in early G1 phase. *Exp Cell Res* **204**: 147–155

Stryer L (1988) *Biochemistry*. WH Freeman: New York

Sugano T, Nitta M, Ohmori H and Yamaizumi M (1995) Nuclear accumulation of p53 in normal human fibroblasts is induced by various cellular stresses which evoke the heat shock response, independently of the cell cycle. *Jpn J Cancer Res* **86**: 415–418

© Cancer Research Campaign 1998

Templeton DJ (1992) Nuclear binding of purified retinoblastoma gene product is determined by cell cycle-regulated phosphorylation. *Mol Cell Biol* **12**: 435–443
Vaupel P, Kallinowski F and Okunieff P (1989) Blood flow, oxygen and nutrient supply, and metabolic microenvironment of human tumors: a review. *Cancer Res* **49**: 6449–6465
Weinberg RA (1995) The retinoblastoma protein and cell cycle control. *Cell* **81**: 323–330
Yamaizumi M and Sugano T (1994) U.v.-induced nuclear accumulation of p53 is evoked through DNA damage of actively transcribed genes independent of the cell cycle. *Oncogene* **9**: 2775–2784
Yao KS, Clayton M and O'Dwyer PJ (1995) Apoptosis in human adenocarcinoma HT29 cells induced by exposure to hypoxia. *J Natl Cancer Inst* **87**: 117–122

 *© Cancer Research Campaign 1998*